# A Tunable, Fullerene‐Based Molecular Amplifier for Vibrational Circular Dichroism

**DOI:** 10.1002/chem.201902190

**Published:** 2019-08-23

**Authors:** Benjamin H. Strudwick, Mark A. J. Koenis, Hans J. Sanders, Valentin P. Nicu, Sander Woutersen, Wybren Jan Buma

**Affiliations:** ^1^ Molecular Photonics Group, Van't Hoff Institute for Molecular Sciences University of Amsterdam Science Park 904 1098 XH Amsterdam The Netherlands; ^2^ Department of Environmental Science, Physics, Physical Education and Sport Lucian Blaga University of Sibiu loan Ratiu Street, Nr. 7-9 550012 Sibiu Romania

**Keywords:** chirality, density functional calculations, fullerenes, spectroelectrochemistry, vibrational circular dichroism

## Abstract

Vibrational circular dichroism (VCD) studies are reported on a chiral compound in which a fullerene C_60_ moiety is used as an electron acceptor and local VCD amplifier for an alanine‐based peptide chain. Four redox states are investigated in this study, of which three are reduced species that possess low‐lying electronic states as confirmed by UV/Vis spectroelectrochemistry. VCD measurements in combination with (TD)DFT calculations are used to investigate (i) how the low‐lying electronic states of the reduced species modulate the amplification of VCD signals, (ii) how this amplification depends on the distance between oscillator and amplifier, and (iii) how the spatial extent of the amplifier influences amplification. These results pave the way for further development of tailored molecular VCD amplifiers.

## Graphical abstract

**Amplifying the difference between left and right with a buckyball**: C_60_ has been used to amplify vibrational circular dichroism signals. Switchable and tunable amplification is achieved by utilizing the various redox states of C_60_. DFT calculations on how vibrational motion influences the electronic distribution pave the way for further optimization of such “extended” amplifiers.

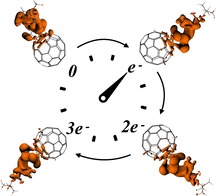

## Introduction

Circular dichroism is a spectroscopic technique sensitive to the chirality of a system that uses the difference between the absorption of left‐ and right‐handed circularly polarised light.^1^ Vibrational circular dichroism (VCD) probes the vibrational transitions of chiral compounds using infrared light.^2^, ^3^, ^4^, ^5^, ^6^, ^7^ The advantage of using vibrational transitions instead of the electronic transitions used in electronic circular dichroism is the increase in resolving power offered by the larger number of transitions that can be accessed. Moreover, vibrational transitions provide a direct fingerprint of the geometrical and electronic structure of the molecule, and are thus inherently more suited to study the stereochemistry, and in particular the absolute configuration and conformational structure, of chiral compounds compared with their electronic counterparts.^3^, ^8^, ^9^, ^10^ These favourable attributes have been utilised in applications ranging from biophysics and pharmaceuticals,^11^, ^12^, ^13^, ^14^, ^15^, ^16^ to enantioselective catalysis^17^, ^18^ and supramolecular compounds.^19^, ^20^, ^21^, ^22^

Despite these favourable characteristics, VCD has one major drawback, which is that the signals are typically 10^−4^–10^−5^ times smaller than the corresponding IR signal. As a result, highly concentrated samples and extensive averaging are required to obtain reliable experimental spectra, even though recent years have also witnessed impressive progress in VCD instrumentation. From the theory underlying VCD^2^, ^4^, ^5^, ^7^ the fundamental reason for the small signals is well understood. The peak intensities in VCD spectra are proportional to the rotational strength^23^ which is given by the imaginary part of the dot product of the electronic and magnetic transition dipole moments. For vibrational transitions the electronic contribution to the total magnetic transition dipole moment is zero within the Born–Oppenheimer (BO) approximation, and one needs to go beyond the BO approximation to recover this contribution:^2^, ^24^

(1) $$\langle \Psi_{f}|{\vec{\mu }}_{mag}^{e}|\Psi_{i}\rangle =\;\left\langle \chi_{v=0}|\sum_{n\neq 0}\frac{\left\langle \psi_{0}|{\vec{\mu }}_{mag}^{e}|\psi_{n}\right\rangle }{E_{n}-E_{0}}\left(\langle \psi_{n}|T_{N}|\psi_{0}\rangle -\langle \psi_{0}|T_{N}\left|\psi_{n}\right\rangle \right)|\chi_{v=1}\right\rangle$$

in which *χ*_*ν*=0_ and *χ*_*ν*=1_ are the nuclear wave functions of the *ν*=0 and *ν*=1 vibrational states in the electronic ground state $\psi_{0}$ , *T_N_* the nuclear kinetic energy operator, ${\vec{\mu }}_{mag}^{e}\;$ the electronic magnetic transition dipole moment, and $\psi_{0}$ and $\psi_{n}$ the BO electronic wave functions for the ground and *n*th electronically excited state with energies *E*_0_ and *E_n_*, respectively. Equation (1) shows that the VCD signal intensities are proportional to the sum of the magnetic transition dipole moments weighted by the associated electronic excitation energy. For closed‐shell organic compounds electronic excitation energies are generally quite high, resulting in small VCD signals. The same equation also shows, however, that compounds with low‐lying electronic states potentially have large and more easily detectable VCD signals.^25^, ^26^, ^27^

Indeed, we have shown in recent years that modulating the electronic structure of a compound of interest may lead to a significant increase of VCD signals. Such a modulation has been achieved with an all‐organic compound by reducing the compound to form a radical anion, and as a result the first electronic transition was lowered by around 16 500 cm^−1^ (neutral→radical anion) and a ten‐fold increase in the VCD signal intensities was achieved.^28^ Even larger amplifications (by more than two orders of magnitude) were achieved by coupling an amino acid or peptide to the low‐lying electronic states of transition‐metal ions.^29^ Interestingly, these studies showed that the signal enhancement strongly depended on the distance between the amplifier and the atoms involved in the mode of interest. This realization was the basis for the subsequent development of switchable amplifiers that could be used as a powerful local probe of chiral structure at user‐defined positions in larger structures.^30^

Up till now, transition metal ions have been used to develop strongly localized “point” VCD amplifiers. One could hypothesize that “extended” amplifiers in which the electronic wavefunctions are delocalized over a larger region of space, and thus are potentially more susceptible to mixing upon vibrational motion, might offer even more favourable amplification properties. Furthermore, the switchable amplifiers used so far can only be switched between an ON and an OFF state. It would be useful to have a “continuously variable” amplifier with several ON positions that each have a different amplification factor.

**Figure 1:**
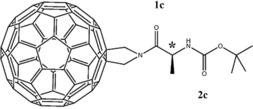
The compound of interest (C_60_‐Ala) which is comprised of a fulleropyrrolidine with alanine substituted onto the pyrrolidine to provide the stereo‐centre (*). The carbonyl vibrational modes **1 c** and **2 c** are probed to investigate amplification by the C_60_ moiety.

From this perspective C_60_ is a compound with attractive characteristics. In solution it can accept up to six electrons.^31^, ^32^ Furthermore, it is a versatile compound from a functionalization point of view^33^, ^34^, ^35^, ^36^, ^37^ and can thus be easily attached to chiral compounds of interest. Studies of the electronic properties of the compound and its reduced states show a lowering of the excitation energies of the lower‐lying electronic states in all of the reduced forms compared with the neutral compound. As a result of these smaller energy gaps one can expect an increased mixing of the lower electronically excited states into the ground state, and thus an enhancement of VCD signals. Moreover, since each of the reduced forms has lower‐lying electronically excited states with different excitation energies, VCD amplification will be adjustable by changing the oxidation state. Here, we explore the VCD amplification properties of C_60_ by experimental and theoretical studies on an amino‐acid‐based chain (alanine) substituted onto fulleropyrrolidine to provide a stereocentre (*) (Figure 1). Alanine was chosen as it typically has small VCD signal intensities that have been studied and amplified before with other amplifiers.^28^, ^29^, ^30^

Previous studies on fullerene–peptide systems^38^ show that electrochemically one can obtain three reduced species for C_60_‐Ala. VCD spectra recorded for the neutral compound and each of these reduced forms will allow us to assess to what extent C_60_ is able to amplify VCD signals and how this amplification depends on the oxidation state. To this purpose we will focus on the carbonyl modes (C=O), labelled **1 c** and **2 c** in Figure 1 that were specifically incorporated to determine the distance dependence of the amplification. These two modes are spectrally isolated from the other modes of C_60_‐Ala and thus enable us to quantify and measure the magnitude of the amplification for single vibrational modes. Furthermore, the literature has reported on these modes before^28^, ^29^, ^30^ allowing for a fair comparison of previous amplifiers with the C_60_ moiety.

## Results and Discussion

**Figure 2:**
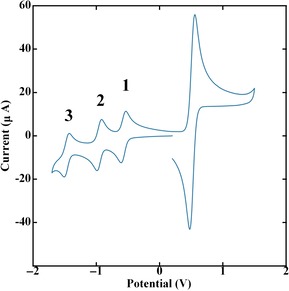
Cyclic voltammetry of (*S*)‐C_60_‐Ala performed on 1.0 mm solutions of the analyte in dichloromethane containing 0.1 m *n*Bu_4_NPF_6_ as the supporting electrolyte. The wave at 0.5 V comes from ferrocene. The three waves between 0 and −2.0 V are associated with $C_{60}^{n-}$ ‐Ala, *n*=1,2,3.

**Figure 3:**
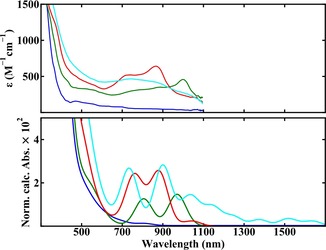
Top: UV/Vis spectra of solutions of (*S*)‐C_60_‐Ala in dichloromethane containing *n*Bu_4_NPF_6_ as the supporting electrolyte at different potentials showing the spectra of C_60_‐Ala (blue), $C_{60}^{-}$ ‐Ala (green), $C_{60}^{2-}$ ‐Ala (red) and $C_{60}^{3-}$ ‐Ala (cyan). Bottom: TD‐DFT predicted electronic spectra of the species shown in the experimental UV/Vis spectrum.

Figure 2 displays the cyclic voltammogram of C_60_‐Ala in DCM which shows three accessible anodic steps at −0.6, −0.9 and −1.45 V. In addition, a peak is observed at +0.5 V which is associated with the oxidation of ferrocene that has been added to the solution for reference purposes. The reduced forms of C_60_‐Ala are easily distinguishable, well separated and fully reversible. For the spectroelectrochemical measurements, potentials were held at −0.75 V (between waves 1 and 2, labelled in Figure 2), −1.25 V (between waves 2 and 3, labelled in Figure 2) and −1.70 V (after the final anodic wave) shifting the equilibrium to produce an excess of $C_{60}^{-}$ ‐Ala, $C_{60}^{2-}$ ‐Ala and $C_{60}^{3-}$ ‐Ala, respectively. Steady‐state UV/Vis measurements of these anionic species and of the neutral form are shown in Figure 3 (top). To compare these spectra with the results of our calculations (vide infra) a wavelength axis is used up to 1800 nm although the employed spectrometer only allowed recording of spectra up to 1100 nm. As expected, these spectra closely resemble the spectra reported previously for neutral and reduced forms of C_60_ albeit that those spectra were recorded in THF^39^, ^40^ whereas we used DCM. For the neutral compound (blue trace) a dominant absorption occurs at 310 nm, but at longer wavelengths weak absorption features are present as well. Within the wavelength range accessible in our experiments the lowest absorption band of $C_{60}^{-}$ ‐Ala (green trace) is found at 1001 nm, close to the absorption bands observed for $C_{60}^{-}$ in the 1000–1100 nm region.^39^, ^40^ The $C_{60}^{2-}$ ‐Ala UV/Vis spectrum (red trace) has two distinct electronic absorption bands at 741 and 865 nm that are similar to the double‐band‐like feature at 829 and 947 nm observed for $C_{60}^{2-}$ .^39^, ^40^ Finally, the UV/Vis absorption spectrum of $C_{60}^{3-}$ ‐Ala (cyan trace) shows a broad feature with a band at 742 nm and a significant shoulder at lower energies around 870 nm. Importantly, from previous studies^39^ it is known that both $C_{60}^{2-}$ and $C_{60}^{3-}$ display a further band around 1300 nm, and we can therefore safely assume that also in the Ala‐substituted species such bands will be present. We thus conclude that upon reduction of C_60_‐Ala a low‐lying electronic manifold becomes available that should allow for amplification of VCD signals.

**Figure 4:**
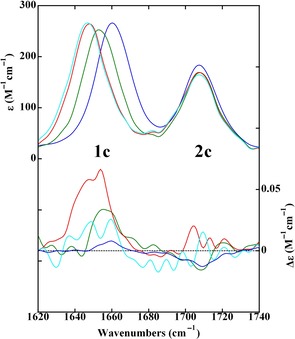
Top panel: FTIR spectra of solutions of (*S*)‐C_60_‐Ala in dichloromethane containing *n*Bu_4_NPF_6_ as the supporting electrolyte at different potentials showing the spectra of C_60_‐Ala (blue), $C_{60}^{-}$ ‐Ala (green), $C_{60}^{2-}$ ‐Ala (red) and $C_{60}^{3-}$ ‐Ala (cyan). Bottom panel: corresponding VCD spectra.

To see how the change in electronic structure affects the normal modes of interest (**1 c** and **2 c**), spectroelectrochemical IR absorption measurements of the carbonyl stretch spectral region were performed. The results are shown in the top panel of Figure 4. For C_60_‐Ala (blue trace) two bands are observed at 1660 and 1707 cm^−1^ in agreement with results obtained for similar systems reported in the literature.^30^ DFT calculations find that these modes are associated with the stretching of the carbonyl group closest to the C_60_ moiety (**1 c**) and the carbonyl group further away (**2 c**). The frequencies and absorption coefficients of **1 c** and **2 c** observed for the various species are given in Table 1. This Table shows that upon reduction, mode **1 c** undergoes a shift to lower frequencies, which is in line with what we would expect on the basis of our previous studies.^28^, ^30^ Mode **2 c**, on the other hand, is in first instance not affected by the change in electronic structure and only starts to show a very minor shift in $C_{60}^{3-}$ ‐Ala. From these observations we conclude that the electronic structure of C_60_ can have a noticeable influence on the carbonyl stretch modes, but that this influence is heavily moderated by the distance from the mode to the amplifier. This conclusion is similar to what has been concluded in Ref. 30, in which it was found that a distance of six bonds in the same peptide chain as used here is too large to be significantly influenced by the amplifier.

**Table 1:** Frequencies (cm^−1^), absorption coefficients (m ^−1^ cm^−1^), differential absorptions (m ^−1^ cm^−1^) and anisotropy factors g (=Δ*ϵ*/*ϵ*) of the carbonyl modes **1 c** and **2 c** for the neutral and various reduced states of (*S*) $C_{60}^{n-}$ ‐Ala. Δ*ϵ*_0_ is the differential absorption of the neutral state (C_60_‐Ala).

Oxidation state	*ν*	*ϵ*	Δ*ϵ*	Δ*ϵ*/*ϵ*×10^3^	Δ*ϵ*/*ϵ*_0_^[a]^
**1 c** C_60_‐Ala	1660	266	0.01	0.04	1.00
$C_{60}^{-}$ ‐Ala	1653	252	0.034	0.13	3.25
$C_{60}^{2-}$ ‐Ala	1647	264	0.066	0.25	6.25
$C_{60}^{3-}$ ‐Ala	1646	266	0.023	0.09	2.25
**2 c** C_60_‐Ala	1707	184	−0.013	−0.07	1.00
$C_{60}^{-}$ ‐Ala	1707	169	−0.016	−0.09	1.29
$C_{60}^{2-}$ ‐Ala	1707	169	0.02	0.12	−1.71
$C_{60}^{3-}$ ‐Ala	1706	164	0.015	0.09	−1.29

[a] Constructed to normalise signals to the neutral state and determine amplification factor.

The bottom panel of Figure 4 displays the VCD spectra of (*S*)‐C_60_‐Ala and its reduced states in the carbonyl‐stretch spectral region, differential absorptions being reported in Table 1. In the IR absorption spectra (top panel) the intensity of the two modes hardly changes, but the VCD signal intensity of **1 c** clearly shows a strong dependency on the oxidation state. Compared to the signal of the neutral form, the signal of the anion is amplified by a factor of about three while the signal of the dianion increases by a factor of about six. The quality of the VCD spectrum of the trianion is poorer than that of the others, probably due to the relatively high potential the solution was held at during the prolonged VCD measurements. Nevertheless, it would appear that the signal of the trianion is still larger than that of the neutral by a factor of about two. The VCD signal intensity of **2 c**, in contrast, shows little to no change. Compared to the neutral species there does appear, however, to be a change in sign of the signal: for the dianion and the trianion positive signals are observed, whereas for the neutral and singly reduced species they are negative. Such a change in sign is indeed also predicted by the VCD calculations on C_60_‐Ala and $C_{60}^{2-}$ ‐Ala (vide infra).

**Figure 5:**
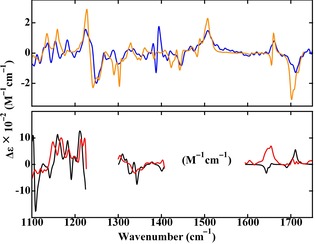
Top: comparison of experimentally recorded (blue) and theoretically predicted (orange) VCD spectra of (*S*)‐C_60_‐Ala. Bottom: comparison of experimentally recorded (red) and theoretically predicted (black) VCD spectra of (*S*)‐$C_{60}^{2-}$ ‐Ala.

The full experimental VCD spectrum of the neutral (*S*)‐C_60_‐Ala species is shown in Figure 5. Comparison with the theoretically predicted spectrum shows in general good agreement and confirms the absolute configuration of the compound. Due to solvent absorption, the experimental and predicted VCD spectra for (*S*)‐$C_{60}^{2-}$ ‐Ala can only be compared in three restricted spectral regions. In agreement with the experiment, the calculations predict that the frequency of **1 c** is lowered upon reduction of the compound. The frequency of **2 c** in the dianion, on the other hand, is calculated to be at higher frequencies than in the neutral, which is not observed in the experiment. More important, however, is that the calculations predict that the VCD signal intensities are enhanced upon reduction and that the sign of **2 c** reverses, although we notice at the same time that the agreement between experiment and theory is in this case not as good as for the neutral species and needs further improvement.

To relate the observed VCD enhancements to the properties of the electronic manifold of C_60_‐Ala in its various oxidation states, we have performed TD‐DFT calculations. Comparison of the theoretically predicted and experimentally recorded UV/Vis absorption spectra (Figure 3, bottom) shows good agreement in the spectral region that is accessible in our experiments. The calculations predict that for $C_{60}^{2-}$ ‐Ala and $C_{60}^{3-}$ ‐Ala, absorptions should be present in the NIR region (*λ*>1100 nm) as well. We do not have access to that region, but spectra recorded previously for C_60_ and its reduced forms^39^, ^40^ indeed indicate the presence of such dipole‐allowed states. When assessing VCD enhancements the importance of the electronic dipole transitions are secondary, instead the magnetic dipole transitions are more influential [see Eq. (1)]. The fact that the UV/Vis absorption spectrum is faithfully reproduced by our calculations leads us to believe that the states involved in such transitions are adequately described by the calculations.

**Figure 6:**
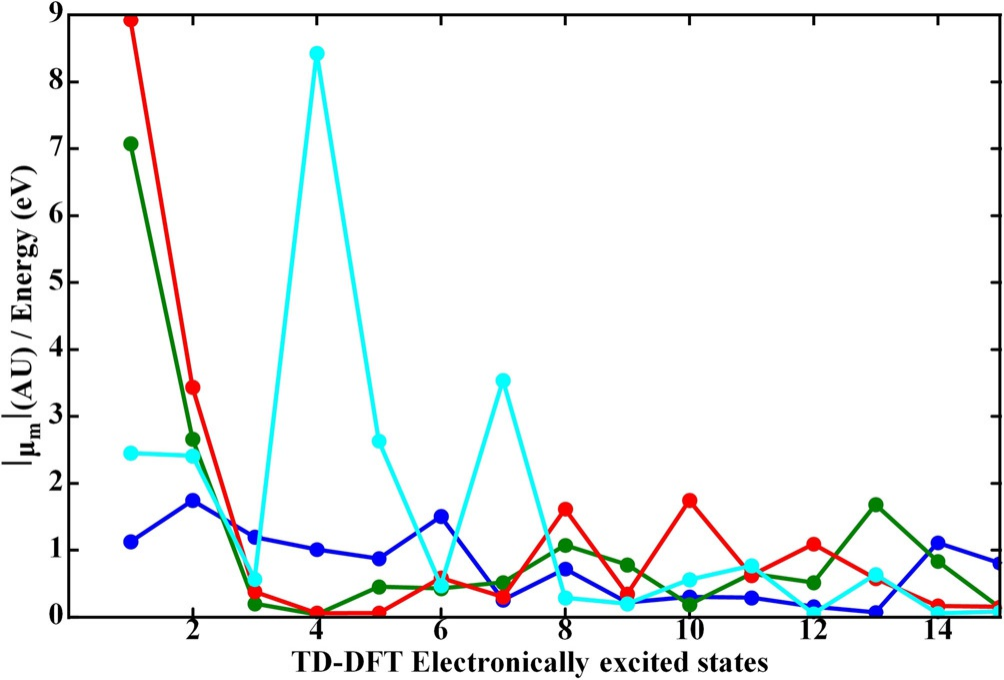
Expansion coefficients $\frac{\left\langle \psi_{0}|{\vec{\mu }}_{mag}^{e}|\psi_{n}\right\rangle }{E_{n}-E_{0}}$ for C_60_‐Ala (blue), $C_{60}^{-}$ ‐Ala (green), $C_{60}^{2-}$ ‐Ala (red) and $C_{60}^{3-}$ ‐Ala (cyan).

Figure 6 shows the expansion coefficients in the sum‐over‐states expression of the lowest 15 electronically excited states for the electronic contribution to the magnetic transition dipole. Although this Figure should only be considered as a qualitative guide, it does lead to a number of relevant observations that support the conclusion that the sum‐over‐states expression appears to capture the main features of the VCD amplification by C_60_. For the neutral species there are no specific states that stand out in terms of providing a larger contribution than other states. For the reduced forms, on the other hand, there are one or two states that have a particularly large contribution. If we focus on these states, we find that these contributions are significantly larger than the contributions observed for the neutral state, which nicely explains the VCD enhancements observed for mode **1 c** of the reduced species. Also, the largest contribution occurs for $C_{60}^{2-}$ ‐Ala, for which the largest amplification factor is observed in the experiments.

**Figure 7:**
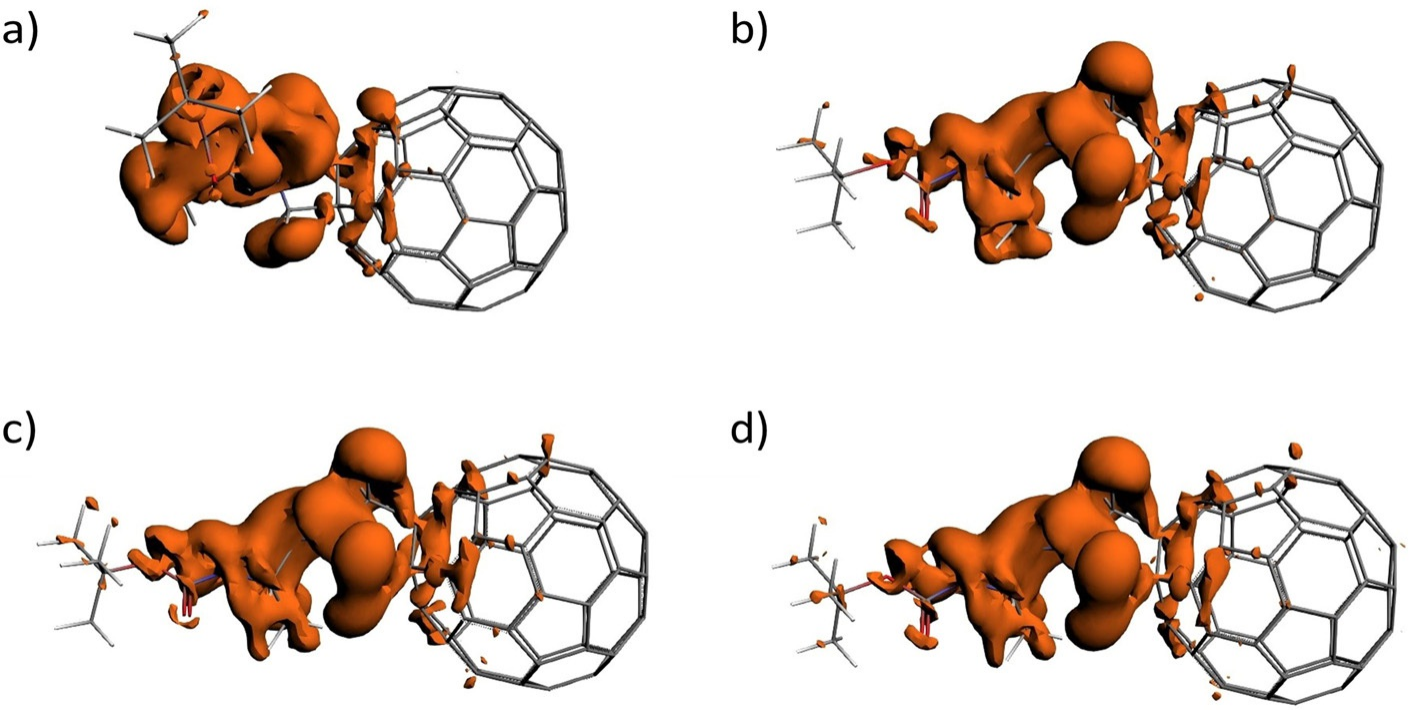
Isosurfaces (−0.0001 a.u.) of SCF electron density differences obtained from calculations on structures in which the carbonyl bond closest to C_60_ has been displaced by 0.3 a.m.u.^1/2^ Å along the normal coordinate and calculations on the equilibrium structure. Panels a, b, c, and d correspond to C_60_‐Ala, $C_{60}^{-}$ ‐Ala, $C_{60}^{2-}$ ‐Ala and $C_{60}^{3-}$ ‐Ala, respectively.

One of the main reasons to investigate the VCD‐amplifying characteristics of C_60_ was the anticipation that (i) larger amplifications might be achieved due to a larger delocalisation of the electronic wavefunctions as compared to amplifiers based on a single metal ion, and (ii) that the different oxidation states of C_60_ would allow for a tuneable amplification. The second objective clearly is achieved in our experiments, but the observed amplification factors definitely do not meet up to our a priori expectations. In order to find out the origin of the poor VCD enhancement, we have determined how perturbing the carbonyl bond closest to the C_60_ moiety by a displacement along the C=O stretch coordinate changes the electron density distribution. Figure 7 shows isosurfaces of the electron‐density difference between the perturbed and unperturbed geometries for the various oxidation states of C_60_‐Ala. Clearly the spatial extent to which the electron density is changed on C_60_ is limited to the part that is closest to the anchor point of Ala, and certainly does not affect the entire electronic distribution of C_60_. Upon reduction to the $C_{60}^{-}$ ‐Ala species an increase in the perturbed electron density and its spatial extent is observed but this increase is relatively small. For the higher‐reduced species a similar increase occurs, yet still only a small fraction remains influenced. With this information at hand it is not so surprising that the amplification of VCD signals is limited, since only a minor part of the amplifier is being used. It is nevertheless gratifying to notice that this increase follows the order anion<trianion<dianion in agreement with the experimental observations that the dianion amplifies most and that the trianion amplifies more than the anion.

## Conclusions

In the present study we have explored the suitability of the reduced forms of C_60_ to serve as a switchable and tuneable amplifier of VCD signals. To this purpose C_60_ has been attached to an alanine‐based peptide chain for which spectroelectrochemical IR absorption and VCD spectra have been recorded. These spectra show that the various oxidation states of C_60_ indeed lead to different VCD amplification factors, but also that amplification critically depends on the distance of the oscillator to the amplifier. Such a distance dependence was previously observed for other amplifying systems.^29^, ^30^ Interestingly, we observe a distance dependence that is quantitatively almost the same despite the fact that the amplifiers differ significantly in size and electronic properties.

While the tunability of the C_60_ amplifier nicely follows expectations based on how the low‐lying electronic‐state manifold is modulated by increasing reduction of C_60_, the magnitude of amplification was found to fall short of a priori ideas that an increased delocalisation of the electronic wavefunction might make it more susceptible to vibronic coupling and thus to an increased mixing of the ground state with electronically excited states. DFT calculations on the changes in the electronic distribution induced by vibrational motion show that the fundamental reason why amplification is not as large as expected is that only a limited part of the amplifier is affected by vibrational motion, that is, the amplifier is not employed to its full spatial extent. It would thus be worthwhile to explore how it might be possible to involve a larger part of C_60_. In the same vein, chemical motifs with an electronic distribution more susceptible to small perturbations would be worth to pursue as potential VCD amplifiers.

## Experimental Section

### Experimental

The full description of the synthesis of (*S*)‐C_60_‐Ala is reported in the Supporting Information (Section S1). All samples were prepared in dichloromethane (DCM) dried over 4 Å molecular sieves to produce a 10 mm solution of C_60_‐Ala and 0.1 m solution of *n*Bu_4_NPF_6_. DCM was chosen as the solvent because of the solubility of C_60_‐Ala and the lack of IR features of DCM in the spectral region of interest. Samples were prepared under an inert atmosphere of dry nitrogen and bubbled with dry argon prior to the measurements. For the UV/Vis and FTIR spectroelectrochemical measurements a standard wire‐grid OTTLE cell was used.^41^ An optically transparent thin‐layer electrochemical VCD‐OTTLE cell was used for the VCD measurements.^42^ Spectra were obtained from averaging three consecutive 20 minute scans. A Bruker Vertex 70 FTIR spectrometer was used for the IR absorption measurements while the VCD measurements were performed using a Bruker PMA 50 VCD module in combination with this Bruker Vertex 70 FTIR spectrometer. Steady‐state UV/Vis absorption spectra were recorded using a HP 8453 UV/Vis absorption spectrometer.

### Cyclic voltammetry

Cyclic voltammetry was performed on 1.0 mm solutions of the analyte in DCM containing 0.1 m *n*Bu_4_NPF_6_ as the supporting electrolyte. The voltammograms were recorded using a PGSTAT302N potentiostat (Metrohm/Autolab), a glassy carbon disk (3.0 mm diameter) as a working electrode, a platinum coil as an auxiliary electrode and a leakless Ag/Ag^+^ reference electrode (eDAQ ET069). To convert the potential values of the Ag/Ag^+^ reference to Fc/Fc^+^ a correction factor (roughly 0.5 V) was used as determined by cyclic voltammetry of ferrocene in DCM using the same reference electrode. At the end of each experiment, ferrocene was added to the solution to check for a possible drift of the reference electrode. All cyclic voltammetric experiments were iR compensated to about 90 % of solution resistance.

### (TD)DFT calculations

A conformational search of C_60_‐Ala was performed with Macromodel from the Schrödinger software suite which led to 26 conformations.^43^, ^44^ These conformations were subsequently optimized with DFT at a BP86/TZP level of theory using the QUILD optimization routines in the ADF2017 software suite.^45^, ^46^ The DFT optimizations were performed separately for the neutral (C_60_‐Ala), anionic ($C_{60}^{-}$ ‐Ala), dianionic ($C_{60}^{2-}$ ‐Ala) and trianionic ($C_{60}^{3-}$ ‐Ala) systems. The calculations on the neutral and dianionic system have been performed with restricted DFT and a spin multiplicity of 1, while calculations on the anionic and trianionic systems used unrestricted DFT and a spin multiplicity of 2. For the optimized conformers vibrational absorption and VCD spectra have been calculated at the same level of theory^47^ albeit that only for the neutral and dianion VCD spectra could be obtained since ADF does not support VCD calculations using unrestricted DFT. For comparison with the experiments the calculated spectra were constructed by convoluting stick spectra with a Lorentzian function with a FWHM of 8 cm^−1^ and weighting the contributions of the individual conformations using an algorithm recently developed in our group.^48^

For the lowest‐energy conformations TD‐DFT calculations have been performed to obtain the vertical excitation energies of the lower‐lying electronic manifold (300 states). To avoid crashes, the dianionic and trianionic electronic calculations were run using the “integeraufbau” and “allow poshomo” options. In addition, a CD spectrum has been calculated to obtain the magnetic dipole transition moments. The electronic absorption spectra have been convoluted with a Gaussian function with a width of 100 nm.

### Associated content

The Supporting Information contains the synthesis of the compound of interest and the full experimental VCD spectrum of all the redox states.

## Conflict of interest

The authors declare no conflict of interest.

## Supporting information

As a service to our authors and readers, this journal provides supporting information supplied by the authors. Such materials are peer reviewed and may be re‐organized for online delivery, but are not copy‐edited or typeset. Technical support issues arising from supporting information (other than missing files) should be addressed to the authors.

Supplementary
