# Supplementary material for: A Tunable, Fullerene‐Based Molecular Amplifier for Vibrational Circular Dichroism
Source: Chemistry. 2019 Aug 23;25(54):12560–6. doi: 10.1002/chem.201902190 (PMC6790965; doi:10.1002/chem.201902190)
Supplement: Supplementary file 1 — Supplementary [file CHEM-25-12560-s001.pdf]

CHEMISTRY
A European Journal

Supporting Information

A Tunable, Fullerene-Based Molecular Amplifier for Vibrational Circular Dichroism

Benjamin H. Strudwick,*[a] Mark A. J. Koenis,[a] Hans J. Sanders,[a] Valentin P. Nicu,[b] Sander Woutersen,*[a] and Wybren Jan Buma*[a]

chem_201902190_sm_miscellaneous_information.pdf

A tunable, Fullerene-based molecular amplifier for Vibrational Circular Dichroism (Supporting Information)

Benjamin H. Strudwick,*† Mark A.J. Koenis,† Hans J. Sanders,† Valentin P. Nicu,‡ Sander Woutersen,*† Wybren Jan Buma*†

*b.h.strudwick@uva.nl; s.woutersen@uva.nl; W.J.Buma@uva.nl

†Molecular Photonics Group, Van 't Hoff Institute for Molecular Sciences, University of Amsterdam, Science Park 904, 1098 XH Amsterdam, The Netherlands

‡Lucian Blaga University of Sibiu, Department of Environmental Science, Physics, Physical Education and Sport, loan Ratiu Street, Nr. 7-9, 550012 Sibiu, Romania

Supporting Information

Tabel of Contents

S1. Synthesis of C_{60} −Ala

S2. Full VCD spectra

S1. Synthesis of C_{60} –Ala

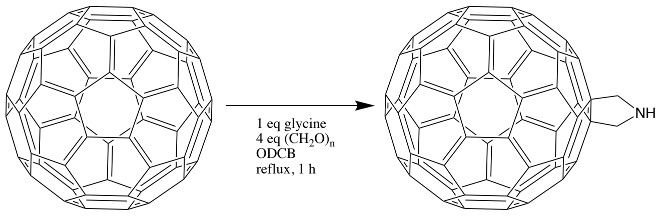

1 **2**

Figure S1: Synthesis of **2**.

Reagents were used as purchased from commercial suppliers. All reactions were carried out under a nitrogen atmosphere. Flash column chromatography was performed using silicagel 60 (230-400 mesh). The following reaction is shown in **Figure S1**: To a solution of **1** (50 mg, 69 µmol) in 1,2-dichlorobenzene (10 ml) glycine (10.4 mg, 138 µmol) and paraformaldehyde (8.3 mg, 276 µmol) was added. The mixture was degassed by bubbling argon through the solution for 10 minutes, after which the mixture was stirred under reflux for 1 hour. TLC indicated partial conversion. The reaction mixture was cooled and transferred to a flash column (SiO_2) immediately. The unreacted C_{60} was eluted with toluene (purple band), after which **2** was eluted with EtOAc/toluene - 25/75 (single brown band). The fraction containing **2** was concentrated to about 25 %: solution **A** (ca. 25 ml).

The following reaction is shown in **Figure S2**: To a cooled (0 ^{o}C) solution of **3** (26 mg, 138 µmol) in CH_2Cl_2 (1 ml) dicyclohexylcarbodiimide (13.7 mg, 66 µmol) was added and the reaction mixture was stirred at 0 ^{o}C for 0.5 h. After this the mixture (a white suspension) was aspirated with a syringe and simultaneously filtered with the help of a syringe filter while being added to cooled (0 ^{o}C) solution **A**. To this triethylamine (10 µl, 7 mg, 69 µmol) was added. This mixture was stirred at RT for 17 h under exclusion of light. TLC indicated partial conversion. The reaction mixture was concentrated to about 75 % and transferred to a flash column (SiO_2) immediately and eluted with a gradient of EtOAc/toluene - 5/95 to 7.5/92.5 to give 11 mg of **4** (**C_{60} –Ala**, yield 17 %) as a brown solid. **Figure S3**: ^{1}H NMR (400 MHz, $CDCl_3$) 5.63-5.34 (m, 5H), 5.02 (p, J = 6.9, 6.4 Hz, 1H), 1.67 (d, J = 6.9 Hz, 3H), 1.49 (s, 9H);

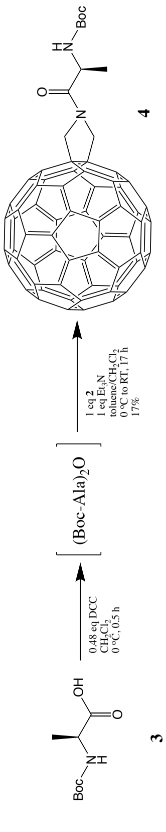

Figure S2: Synthesis of **4** (**C_{60}** −**Ala**).

TLC R_f 0.7 (**4**, EtOAc/toluene - 25/75), R_f 0.4 (**2**, EtOAc/toluene - 25/75).

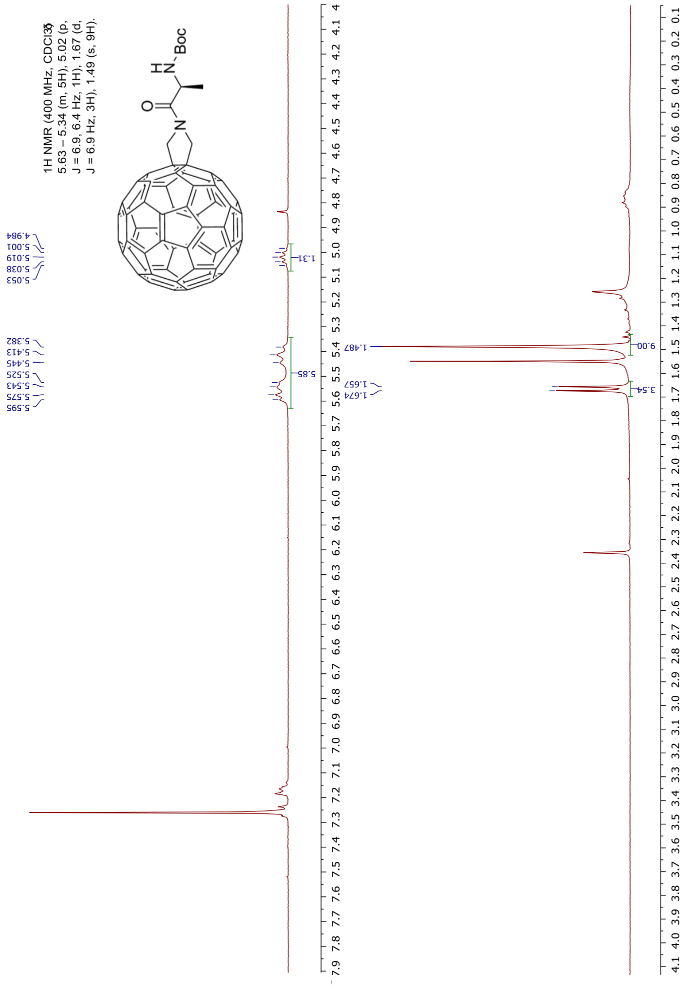

Figure S3: ^{1}H NMR spectrum (recorded on a Bruker Advance ARX 400, 400 MHz) of $\mathbf{C}_{60}$ $-$**Ala** in $CDCl_3$ (purchased from Euriso-top).

S2. Full VCD spectra

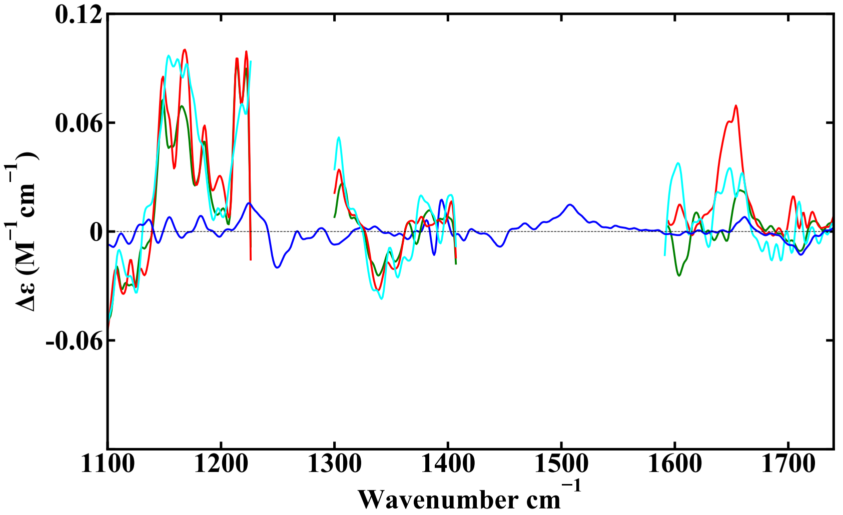

Figure S4: The full experimentally obtained VCD spectrum of all the redox species: C_{60}–Ala (blue), C_{60}^{-}–Ala (green), C_{60}^{2-}–Ala (red) and C_{60}^{3-}–Ala (cyan).
